# Pulmonary Cavitation as a Late and Self-Limited Complication of COVID-19 Pneumonia: A Case Report

**DOI:** 10.7759/cureus.100368

**Published:** 2025-12-29

**Authors:** Manuel Osório, Miguel Silveira

**Affiliations:** 1 Pulmonology, Unidade local de Saude Amadora Sintra, Lisbon, PRT; 2 Pulmonology, Unidade local de Saude Amadora Sintra, Hospital Prof. Dr. Fernando Fonseca, Lisbon, PRT

**Keywords:** bacterial superinfection, covid-19, fungal superinfection, post-acute sequelae of sars-cov-2 infection, pulmonary cavitation

## Abstract

Pulmonary cavitation is an uncommon late complication of coronavirus disease (COVID-19), and its underlying mechanisms remain incompletely understood.

We report a case of a 37-year-old previously healthy man who developed bilateral cavitary lung lesions in the first weeks following hospitalization for severe COVID-19 pneumonia requiring noninvasive ventilation, systemic corticosteroids, and empiric broad-spectrum antibiotics. A computed tomography pulmonary angiogram performed during the index admission showed typical COVID-19 changes without cavitation. Twenty-one days after discharge, he was readmitted with pleuritic chest pain and was found to have a new large, thick-walled cavitary lesion with an air-fluid level in the left lower lobe, followed one week later by a second gas-filled cavity in the right upper lobe. Despite these radiological findings, he remained clinically stable, with no fever, respiratory compromise, or leukocytosis. Extensive evaluation, including repeat computed tomography (CT) pulmonary angiography, blood and sputum cultures, autoimmune screening, bronchoscopy, bronchoalveolar lavage (with bacterial, mycobacterial, and fungal studies), and galactomannan testing, was entirely negative.

In view of the reassuring clinical and laboratory profile, antimicrobial therapy was stopped, and the patient was managed conservatively with close follow-up. Serial imaging over five months demonstrated complete resolution of both cavities without further intervention.

This case illustrates a self-limited form of post-COVID pulmonary cavitation in a young adult with a negative infectious workup and emphasizes the importance of integrating clinical stability, inflammatory markers, and thorough diagnostic assessment to distinguish noninfectious post-COVID cavitation from necrotizing infection and to avoid unnecessary prolonged antimicrobial treatment.

## Introduction

Pulmonary cavitation has a broad differential diagnosis and is classically associated with necrotizing bacterial pneumonia, mycobacterial and fungal infections, granulomatous and autoimmune disease, malignancy, and pulmonary infarction.

Pulmonary cavitation is an uncommon radiological finding in the context of COVID-19 pneumonia. Early imaging descriptions of SARS-CoV-2 infection consistently reported predominant patterns such as bilateral ground-glass opacities, vascular enlargement, interlobular septal thickening, and subpleural bands, while cavitary lesions were not typically observed in the initial stages of disease [1].

As clinical experience accumulated, subsequent observational studies demonstrated that pulmonary cavitation may develop during the subacute or recovery phase of COVID-19. A retrospective follow-up study reported an incidence of approximately 3.4%, and additional case series describe cavitary lesions appearing between the second and 12th week (18 to 82 days) after the initial infection [2-4]. These findings suggest that, although uncommon, post-COVID cavitation has become a recognized manifestation within the broader spectrum of late pulmonary sequelae.

Several mechanisms have been proposed to explain cavity formation after COVID-19. Case series have highlighted the contribution of secondary bacterial and fungal infections, including COVID-associated pulmonary aspergillosis and mucormycosis, particularly among patients treated with high-dose corticosteroids or immunomodulatory agents [3,4].

In parallel, individual case reports have documented cavitary lesions emerging after otherwise uncomplicated COVID-19 pneumonia, in which extensive microbiological and autoimmune evaluations were negative. These observations raise the possibility that direct viral-related parenchymal necrosis or ischemic microangiopathic injury may also play a role in cavity formation [5-7].

Further support for ischemic mechanisms comes from autopsy studies of patients who died from COVID-19, which have demonstrated diffuse alveolar damage, severe endothelial injury, widespread alveolar capillary microthrombi, and marked pulmonary microangiopathy - pathological features that may predispose to localized parenchymal necrosis and, in some cases, subsequent cavitation [8].

Despite these insights, post-COVID cavitation without evidence of superinfection remains poorly defined, particularly in younger patients who are clinically improving at the time the lesions are detected. In such situations, the appearance of a new cavitary lesion typically prompts a comprehensive evaluation to exclude tuberculosis, fungal infection, and lung abscess, with important implications for management and follow-up.

Here, we describe a previously healthy young adult who developed bilateral pulmonary cavities shortly after hospitalization for severe COVID-19 pneumonia, in whom an extensive diagnostic workup was unrevealing and complete radiological resolution occurred without targeted antimicrobial therapy, illustrating a self-limited form of post-COVID cavitation.

This case was previously presented in poster form at the 13th Iberian Pneumology Congress (Coimbra, Portugal; July 7-8, 2022).

## Case presentation

**Figure 1:**
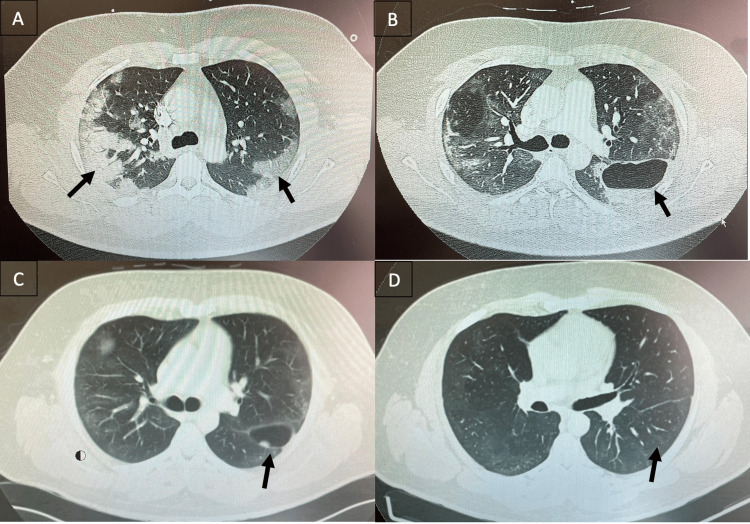
Serial axial chest CT images (lung window) on July 11 (A), August 7 (B), September 11 (C), and December 8 (D), 2021, showing the evolution of post-COVID lung changes. Initial scans demonstrate bilateral parenchymal abnormalities compatible with late-phase COVID-19 pneumonia, followed by the development of a large thick-walled cavitary lesion with an air-fluid level in the left lower lobe and subsequent near-complete radiological resolution with minimal residual changes.

A 37-year-old previously healthy man was hospitalized in June 2021 with acute SARS-CoV-2 pneumonia. During that admission, he developed acute hypercapnic respiratory failure requiring noninvasive ventilation, systemic corticosteroid therapy, and empiric broad-spectrum antibiotics. A computed tomography (CT) pulmonary angiogram performed during this hospitalization excluded pulmonary embolism and demonstrated bilateral peripheral consolidations predominantly in the lower lobes, with upper-lobe ground-glass opacities typical of COVID-19 pneumonia (Figure 1, Panel A). No cavitary lesions were identified at that time, and his clinical condition progressively improved, allowing discharge from the hospital.

Twenty-one days after discharge, in July 2021, he re-presented to the emergency department with new-onset, left-sided pleuritic chest pain. He denied fever, cough, sputum production, hemoptysis, or other systemic symptoms. On examination, he was afebrile and hemodynamically stable, maintaining normal oxygen saturation on room air. Laboratory testing revealed an elevated D-dimer (4365 ng/mL) and a mildly increased C-reactive protein (2.75 mg/dL), without leukocytosis or other relevant abnormalities. In view of the pleuritic chest pain and raised D-dimer, a repeat CT pulmonary angiogram was performed. Pulmonary embolism was again excluded. However, a new thick-walled, fluid-containing cavitary lesion was identified in the left lower lobe, measuring approximately 80 × 45 mm, with a clear air-fluid level (Figure 1, Panel B). The surrounding parenchyma showed residual abnormalities consistent with late or fibrotic-stage COVID-19 pneumonia. A diagnosis of probable lung abscess secondary to necrotizing infection was initially considered, and empiric intravenous antibiotics were commenced.

During the subsequent hospital stay, the patient remained clinically stable, apyretic, and without respiratory compromise. Serial laboratory tests consistently showed low inflammatory markers, with no rise in leukocyte counts. A comprehensive diagnostic workup was undertaken, including blood cultures, sputum cultures, autoimmune screening, and fungal studies, all of which were negative.

A follow-up chest CT performed seven days later demonstrated persistence of the left lower lobe cavitary lesion and revealed a new, well-defined, gas-filled cavity in the right upper lobe, measuring 40 × 37 mm, surrounded by parenchymal abnormalities compatible with organizing pneumonia.

Flexible bronchoscopy showed normal airways without purulent secretions or endobronchial lesions. Bronchial washings and bronchoalveolar lavage (BAL) samples were negative for bacterial, mycobacterial, and fungal pathogens. Acid-fast bacilli smear and culture were negative, and BAL galactomannan testing was also negative. Cytological analysis revealed no malignant cells. Comprehensive laboratory, microbiological, bronchoalveolar lavage, and autoimmune findings from the initial admission and the subsequent re-admission are summarized in Table 1.

**Table 1:** Laboratory, microbiological, and immunological findings

Parameters	Initial admission for COVID-19 pneumonia	Re-admission with a cavitary lung lesion	Units/Result	Reference range/Expected result
Hematology				
Hemoglobin	13.6	13.4	g/dL	13.0-17.0
White blood cell count	14.1	9.2	×10⁹/L	4.0-11.0
Neutrophils	6.1 (78%)	7.1 (77%)	×10⁹/L (%)	1.8-7.5 (40-75)
Lymphocytes	0.9 (12%)	0.9 (10%)	×10⁹/L (%)	1.0-4.0 (20-45)
Platelets	280	350	×10⁹/L	150-400
Inflammatory and coagulation markers				
C-reactive protein (CRP)	15.8	2,75	mg/dL	<0.5
D-dimer	1320	4365	ng/mL	<500
Fibrinogen	650	690	mg/dL	200-400
International normalized ratio (INR)	1.08	1.10	-	0.8-1.2
Renal and liver function				
Creatinine	0.9	0.8	mg/dL	0.7-1.2
Urea	32	34	mg/dL	10-50
Aspartate aminotransferase (AST)	30	32	U/L	<40
Alanine aminotransferase (ALT)	28	29	U/L	<41
Alkaline phosphatase	90	95	U/L	44-147
Total bilirubin	0.7	0.8	mg/dL	0.2-1.2
Lactate dehydrogenase (LDH)	380	221	U/L	135-225
Arterial blood gas (on room air)				
pH	7.28	7.41	-	7.35-7.45
PaCO₂	59	38	mmHg	35-45
PaO₂	52	78	mmHg	80-100
HCO₃⁻	28,2	24	mmol/L	22-26
Lactate	2.1	1.2	mmol/L	0.5-2.0
PaO₂/FiO₂ ratio	248	371	-	>300
Microbiology				
Blood cultures (2 sets)	Not performed	No growth at 5 days	Qualitative	-
Sputum culture	Not performed	Normal respiratory flora; no pathogenic bacteria isolated	Qualitative	-
Bronchoalveolar lavage (BAL) microbiology and galactomannan				
BAL bacterial culture	-	No pathogenic bacteria isolated	Qualitative	-
BAL mycobacterial smear and culture	-	Negative smear; no growth on culture	Qualitative	-
BAL fungal culture	-	No fungal growth	Qualitative	-
BAL galactomannan	-	Index 0.20 (negative)	Index value	Negative (<0.5)
Cytology and pathology				
BAL cytology	-	No malignant cells; neutrophil-predominant inflammatory pattern	Descriptive	-
Autoimmune/immunologic workup				
Antinuclear antibodies (ANA)	-	Negative	Qualitative	-
Antineutrophil cytoplasmic antibodies (ANCA)	-	Negative (p-ANCA and c-ANCA)	Qualitative	-
Rheumatoid factor	-	Negative	IU/mL	<20
Anticyclic citrullinated peptide (anti-CCP)	-	Negative	U/mL	<20
Serum IgE	-	60	IU/mL	<100
Complement C3/C4	-	155/31	mg/dL	100-233/14-48

In the absence of clinical deterioration, with persistently low inflammatory markers and a completely negative microbiologic and autoimmune evaluation, the overall picture was considered most consistent with post-COVID pulmonary cavitation rather than active necrotizing infection. Antimicrobial therapy was therefore discontinued, and the patient was managed expectantly.

He was discharged with a planned close outpatient follow-up. Serial chest CT scans obtained between August and December 2021 demonstrated progressive reduction in the size of both cavitary lesions (Figure 1, Panel C), culminating in complete radiological resolution without the need for further specific intervention (Figure 1, Panel D). Throughout this follow-up period, the patient remained asymptomatic, with no recurrence of chest pain, fever, or respiratory symptoms.

## Discussion

Although cavitation is not a typical radiological feature of acute SARS-CoV-2 infection, accumulating evidence shows that it may develop as a delayed pulmonary sequela. Initial CT-based descriptions of COVID-19 emphasized non-cavitary patterns, but later clinical experience revealed that cavities can appear during the recovery phase in a small subset of patients [1-4]. Reported series indicate that these lesions tend to arise several weeks after the acute illness, with published intervals ranging from approximately two to 12 weeks following symptom onset [2-4]. The presentation of our patient, with the first cavity identified three weeks after discharge for severe COVID-19 pneumonia, aligns closely with this emerging temporal pattern.

A retrospective follow-up study further showed that post-COVID cavitation can develop across varying degrees of disease severity. Although most cases occurred in patients recovering from moderate-to-severe COVID-19, two individuals with a mild clinical course and no respiratory failure also developed sizable cavitary lesions during convalescence, indicating that this complication is not limited to severe disease [2]. Furthermore, evidence from an intensive care cohort shows that pulmonary cavitation is considerably more frequent among critically ill patients, with an incidence of approximately 11% in those requiring ICU-level support for severe COVID-19 [9].

The mechanisms underlying post-COVID cavitation appear to be multifactorial. Several case series have highlighted the role of secondary infection, particularly necrotizing bacterial pneumonia and angioinvasive fungal disease. Documented pathogens include *Staphylococcus aureus*, *Haemophilus influenzae*, and *Klebsiella pneumoniae*, as well as opportunistic fungal infections such as COVID-associated pulmonary aspergillosis (CAPA) and mucormycosis, frequently in patients treated with high-dose corticosteroids or immunomodulatory agents [3,4,10].

A case of post-COVID cavitation attributed to bacterial superinfection has also been described, demonstrating how secondary pathogens can precipitate necrotizing parenchymal injury and contribute to the development of cavitary lesions [10]. Additional series have described extensive cavitary disease in the setting of post-COVID necrotizing infection, further reinforcing the destructive potential of severe superinfection in susceptible individuals [11].

However, post-COVID cavitation cannot be fully explained by secondary infection alone. Multiple reports describe cavitary lesions occurring after an otherwise uncomplicated viral illness, with consistently negative bacterial, mycobacterial, fungal, and autoimmune investigations [5-7]. In these cases, as in our patient, the absence of fever, leukocytosis, or rising inflammatory markers, together with sterile bronchoalveolar lavage and a negative galactomannan assay, strongly argues against ongoing necrotizing infection.

These findings have led several authors to propose that direct viral-related parenchymal injury, possibly potentiated by microangiopathic changes, may contribute to cavity formation in selected patients [5,7]. This mechanism offers a plausible explanation for cavitation arising in clinically stable individuals with no evidence of superinfection, complementing the infectious pathways suggested in other series.

Growing evidence suggests that microangiopathic injury may contribute to the pathogenesis of post-COVID cavitation. Microthrombotic phenomena and ischemic damage have been proposed as potential explanations for cavitary changes in patients without microbiological evidence of infection [6]. Autopsy data further support this hypothesis, demonstrating diffuse alveolar damage, severe endothelial injury, widespread thrombosis with microangiopathy, and abundant alveolar capillary microthrombi in fatal COVID-19 [8], a pattern consistent with a microangiopathic process that could plausibly promote localized ischemia, microinfarction, and, in selected cases, cavitation.

A detailed report of a previously healthy young adult who developed cavitary lung disease several months after mild COVID-19 described a necrotizing cavitary lesion with dense acute inflammation, granulation tissue, and no detectable microorganisms on surgical pathology [12], indicating that delayed post-COVID cavitation can arise in structurally normal lungs and may not always be attributable to overt secondary infection.

The diagnostic approach in this setting must be systematic and comprehensive. The emergence of a cavitary lesion soon after COVID-19 warrants thorough evaluation to exclude pulmonary embolism, abscess, tuberculosis, and fungal disease, given their distinct prognostic and therapeutic implications. In our case, the workup included two CT pulmonary angiograms, broad microbiological testing, autoimmune screening, bronchoscopy, and BAL analysis, all of which were negative.

Similar structured evaluations have been reported in other series, underscoring the importance of excluding treatable causes while avoiding unnecessary exposure to prolonged antimicrobials when evidence does not support active infection [5-7].

Our case contributes to the characterization of the subgroup of patients who develop post-COVID cavitation with a completely negative infectious evaluation and a benign, self-limited course.

While many reported cases involve older patients or those with immunosuppression, diabetes, or significant coinfection, our patient remained clinically stable, with persistently low inflammatory markers and full radiological resolution between August and December 2021 under conservative management alone. This aligns with reports of spontaneous improvement in select patients, suggesting that not all post-COVID cavitations require aggressive antimicrobial therapy or surgical intervention [5-7,11,12]. Recognition of this phenotype is essential to prevent overtreatment and guide appropriate follow-up.

Taken together, available evidence suggests that post-COVID pulmonary cavitation is a heterogeneous entity with diverse etiologies, including bacterial or fungal superinfection, post-infectious organizing pneumonia, embolic ischemic parenchymal injury, and direct virus-mediated necrosis. Our case illustrates a self-limited form of this complication and underscores the importance of integrating clinical stability, inflammatory markers, and a comprehensive diagnostic workup to distinguish infectious from noninfectious causes. Further studies are required to better define predisposing factors, long-term outcomes, and the relative contribution of each underlying pathophysiological mechanism.

## Conclusions

Post-COVID pulmonary cavitation is an uncommon complication that demands careful exclusion of alternative infectious and thromboembolic causes. This case illustrates that, in a clinically stable patient with declining inflammatory markers and a negative extensive workup, spontaneous resolution is possible, and close radiological surveillance may be a reasonable strategy.
